# Awareness and Level of Knowledge About Surgical Site Infections and Risks of Wound Infection Among Medical Physicians in King Abdulaziz University Hospital: Cross-Sectional Study

**DOI:** 10.2196/12769

**Published:** 2019-03-06

**Authors:** Wahbi Albishi, Marwan Ahmad Albeshri, Hatan Hisham Mortada, Khaled Alzahrani, Rakan Alharbi, Farrah Aljuhani, Saleh Aldaqal

**Affiliations:** 1 College of Medicine, King Abdulaziz University Jeddah Saudi Arabia; 2 Department of Surgery, King Abdulaziz University Hospital Jeddah Saudi Arabia

**Keywords:** surgical site infections, knowledge, attitude, infection

## Abstract

**Background:**

Surgical site infections (SSIs) are one of the leading causes of death, and its prevention is a key element of applying the concept of patient safety and quality care.

**Objective:**

This study aimed to assess the level of knowledge about SSIs and risks of wound infection among medical physicians in King Abdulaziz University Hospital.

**Methods:**

All surgical and medical consultants, specialists, residents, and medical interns were invited to participate in the study. A 20-Item multiple-choice questionnaire was developed by reviewing the previous literature and with the help of a group of certified surgeons to assess the level of knowledge in all participants.

**Results:**

A total of 119 doctors were included in this study. Among all respondents, 92 (77.3%) were intern doctors, 16 (13.4%) were resident doctors, and 11 (9.2%) were specialist doctors. Moreover, 66 (55.5%) doctors knew the definition of SSI. Only one-quarter, that is, 30 (25.2%) doctors knew about the incidence of SSI. In addition, 8 doctors (6.7%) had good knowledge, 75 (63.0%) had fair knowledge, and 36 (30.2%) had poor knowledge regarding SSI according to this study.

**Conclusions:**

Level of knowledge about SSIs and risks of wound infections among medical physicians should be improved to ensure better wound care and quality care for the patients.

## Introduction

### Background

Health care–associated infections (HCAIs) are one of the leading causes of death that involve a huge number of patients every year worldwide [1]. Surgical site infection (SSI) accounts for more than 20% of HCAIs, and it is the most frequent HCAI in low-income and middle-income countries [1,2]. A study conducted in England, Wales, Northern Ireland, and the Republic of Ireland among 75,694 patients showed that the overall prevalence of HCAIs was 7.59%, with SSI accounting for 14.5% of these infections [3]. According to the World Health Organization and the Centers for Disease Control and Prevention (CDC), SSIs are considered one of the most preventable HCAIs [4]. SSI can be prevented by many infection control methods including surgical hand preparation, enhanced nutritional support, preoperative bathing, surgical site skin preparation, hair removal, mechanical bowel preparation, and the use of oral antibiotics [1,5]. Proper hand hygiene and the correct use of basic precautions during invasive procedures are simple and of low cost but require staff education and surveillance systems [5]. As known, SSI prevention is the key element of applying the concept of patient safety and quality care [6]. In the literature, most of the studies described the level of knowledge and awareness of SSI among nurses but not doctors [7]. Although nurses play an important role in preventing SSI, interns, residents, specialists, and consultants are also involved in patient care, and their role should be studied.

### Objective

In this questionnaire-based survey, we aimed to analyze the current awareness and level of knowledge about SSI and risks of wound infections among physicians (interns, residents, specialists, and consultants) in King Abdulaziz University Hospital (KAUH), Jeddah, Saudi Arabia.

## Methods

### Study Design and Participants

We conducted this hospital-based cross-sectional study at the Department of Surgery at KAUH between January 2018 and June 2018. All surgical and medical consultants, specialists, residents, and medical interns were invited to participate in the study. KAUH is located in Jeddah, Saudi Arabia, which is the second largest city in the kingdom of Saudi Arabia. It is a tertiary care center with a bed capacity of 876 including inpatient beds, intensive care unit beds, and emergency rooms.

There were a total of 450 interns working at the hospital at the time of this study, and all of them were invited to participate in the study. The total number of consultants and specialists working at the hospital at the time of this study could not be estimated. However, the questionnaire was distributed to all of the included departments by the departments’ coordinators and secretaries.

A 20-item multiple-choice questionnaire was developed by reviewing the previous literature and with the help of a group of certified surgeons to identify the important factors and knowledge that must be followed to reduce SSIs and to develop a valid questionnaire to properly assess the level of knowledge about SSIs and risks of wound infection among physicians in KAUH. The questionnaire was distributed randomly among the included sample.

After recruitment, the participants were asked to specify their position. After that, the questionnaire was distributed among the participants, and they were asked to answer the questions according to their knowledge about SSIs and risks of wound infections. Then, the level of knowledge was assessed for all participants.

Ethical approval was obtained from the Department of Bioethics at KAUH before the start of the study.

### Statistical Analysis

Job position and answers to the SSI-related questions were presented as frequencies and percentages. One point was assigned for each of the correct answers to 20 SSI-related questions and 0 point was given for wrong answers. So, the obtainable points range between 0 (if all answers were wrong) and 20 (if all answers were correct). The mean (SD) knowledge score for all respondents and categorized by job position were presented. Furthermore, the respondents were categorized as having good knowledge (for ≥80% correct answers), fair knowledge (for 50%-79% correct answers), and poor knowledge (for <50% correct answers). The analysis was performed with 95% CI using Statistical Package for the Social Sciences (SPSS), version 23 (IBM, Armonk, NY, USA).

## Results

**Figure 1:**
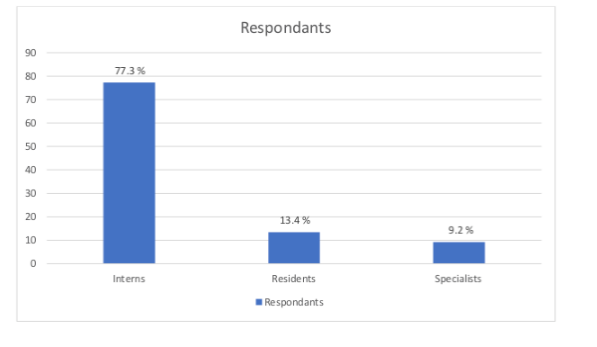
Distribution of all respondents by their position (n=119).

A total of 119 doctors were included in this study. Among all respondents, 92 (77.3%, 92/119) were intern doctors, 16 (13.4%, 16/119) were resident doctors, and 11 (9.2%, 11/119) were specialist doctors (Figure 1).

Moreover, 66 (55.5%, 66/119) doctors knew the definition of SSI according to the US CDC. Almost half (50.4%, 60/119) of the doctors knew that the superficial SSI meant an infection of the skin and subcutaneous tissue and 58.8% knew that the superficial SSI was responsible for more than half of all SSIs. A total of 47 respondents (39.5%, 47/119) knew about the most common organisms causing SSI: *Staphylococcus* *aureus* and *Escherichia coli*. Most of the respondents (78.2%, 93/119) knew about the best time for administering prophylactic antibiotics, which was within 1 hour before surgery. Only one-quarter, that is, 30 (25.2%, 30/119) doctors, knew about the incidence of SSI, which was 1% to 3%. A total of 39.5% of study participants identified fidaxomicin as an antibiotic that was not commonly recommended for SSI. Moreover, 58 (48.7%, 58/119) of the respondents knew that a clean-contaminated wound was defined as an incision under sterile condition with an incision into a hollow viscus with no active infection. Less than half (46.2%, 55/119) of the doctors knew that the hairy skin was less likely to be associated with SSI. The majority (84.0%, 100/119) of the doctors knew about the complications of SSI, whereas about one-third (34.5%, 41/119) of the doctors knew about the correct recommendations for preoperative SSI prevention. A total of 89.1% (106/119) of the doctors answered that the infected wounds could exhibit purulent pus. About half of them stated the correct time for hair removal, but only 22.7% (27/119) knew about the preferred hair removal method, which was by clipping. Steroid use association with SSI was known by 83.2% (99/119) doctors. Furthermore, 83 (69.7%, 83/119) doctors answered that the serum albumin level was the most commonly used marker to assess nutritional status. The fourth step in hand hygiene was correctly stated by the relatively small number of doctors (20.4%, 24/119), but most of them knew about the first step (69.7%). In addition, 80.7% (96/119) of doctors correctly answered that the purpose of preoperative skin cleansing was to reduce the burden of skin flora, thus reducing the risk of SSI (Multimedia Appendix 1).

A total of 8 doctors (6.7%, 8/119) had good knowledge, 75 (63.0%, 75/119) had fair knowledge, and 36 (30.2%, 36/119) had poor knowledge regarding SSI according to this study. The mean (SD) knowledge score of all respondents was 10.93 (SD 3.24). The mean (SD) knowledge score of interns, residents, and specialists was 10.40 (SD 3.10), 12.13 (SD 3.70), and 13.64 (SD 1.69), respectively (Tables 1 and 2).

**Table 1:** Distribution of all by knowledge level regarding surgical site infections and risks factors for wound infection in 119 physician respondents at King Abdul-Aziz University Hospital between January and June 2018 (n=119).

Level of Knowledge	Score
Good knowledge (≥80% correct answer), n (%)	8 (6.7)
Fair knowledge (50%–79% correct answer), n (%)	75 (63.0)
Poor knowledge (<50% correct answer), n (%)	36 (30.2)
Mean knowledge score of all respondents, mean (SD)	10.93 (3.24)

**Table 2:** Distribution of mean knowledge score of all respondents by their job position (n=119).

Job position	Mean knowledge score, mean (SD)
Intern	10.40 (3.10)
Resident	12.13 (3.70)
Specialist	13.64 (1.69)

## Discussion

### Principal Findings

SSI is defined by the CDC as a proliferation of the causative micro-organisms, which can be superficial (colonization in an incisional site within the skin or subcutaneous fat), deep (colonization in musculofascial layers), or in an organ or cavity [7]. Between 3% to 5% of all the patients who undergo surgery will experience SSI, as mentioned in the literature [8]. The incidence of SSI is 15 times higher in unindustrialized countries than in industrialized countries, for example, 38% in Nigeria [3], 12% in India [9], and 19% in Ethiopia [10]. A study conducted in India in 2016 mentioned that the perception of health care staff about SSIs was good enough (94%), but the practices were inadequate (47%) [11]. On the other hand, a study conducted in Bangladesh reported that the perception of majority of nurses (70%) was inadequate regarding the SSI prevention, but the practices of most of the nurses were higher (98.3%) [12]. However, in the literature, most of the studies described the level of knowledge and awareness of SSI among nurses but not doctors. Although nurses play an important role in preventing SSI, interns, residents, specialists, and consultants are also involved in patient care, and their role should be studied. In this questionnaire-based survey, we analyzed the current awareness and level of knowledge about SSI and risks of wound infections among physicians (interns, residents, specialists, and consultants) in KAUH.

A total of 119 doctors were included in this study. A similar number of participants were reported in different studies [4,5,13]. Most of the study participants (77.3% 92/119) were intern doctors, which is comparable with a study conducted by Patil et al on the prevention of SSI [4]. A total of 55.5% (66/119) of doctors knew the definition of SSI according to the US CDC. In contrast to a study conducted by Labeau et al, which showed that only 7% of nurses knew the correct classification of SSI [14], in our study, almost half of the doctors knew that the superficial SSI means an infection of the skin and subcutaneous tissue and 58.8% (70/119) knew that the superficial SSI is responsible for more than half of all SSIs.

A total of 47 respondents (39.5% 47/119) knew about the most common organisms causing SSI (*S aureus* and *E coli*). Most of the respondents (78.2% 93/119) knew about the best time for administering prophylactic antibiotics, which was within 1 hour before surgery. A lower rate (57.58%) was observed in a study conducted in Nigeria among doctors and nurses to assess the knowledge and infection control practices [11].

Only one-quarter (30/119 doctors) knew about the incidence of SSI, which was 1% to 3%. A total of 39.5% (39/119) of the study participants identified fidaxomicin as an antibiotic, which was not commonly recommended for SSI. Moreover, 58/119 (48.7%) of the doctors knew that a clean-contaminated wound was defined as an incision under sterile condition with an incision of a hollow viscus with no active infection. Less than half (46.2% 55/119) of the doctors knew that the hairy skin was less likely to be associated with SSI.

The majority (84.0% 100/119) of the doctors knew about the complications of SSI, whereas about one-third (34.5% 41/119) of the doctors knew about the correct recommendations for preoperative SSI prevention. A total of 89.1% (106/119) of the doctors answered that the infected wounds can exhibit purulent pus. About half of them stated the correct time for hair removal, but only 22.7% (27/119) knew about the (clipping) preferred hair removal method, in contrast to the study by Labeau et al, which showed that only 26% knew the correct time for hair removal, whereas 50% (60/119) knew that electric clippers were recommended [5]. Steroid use was associated with SSI was known by 83.2% (99/119) doctors. A total of 83/119 (69.7%) doctors answered that the serum albumin level is the most commonly used marker to assess nutritional status.

The fourth step in hand hygiene was correctly stated by the relatively small number of doctors (20.4% 24/119), but most of them knew about the first step (69.7% 83/119). Moreover, 80.7% (96/119) of doctors correctly answered that the purpose of preoperative skin cleansing is to reduce the burden of skin flora, thus reducing the risk of SSI (Multimedia Appendix 1).

A total of 8/119 doctors (6.7%) had good knowledge, 75/119 (63.0%) had fair knowledge, and 36/119 (30.2%) had poor knowledge regarding SSI according to this study. Similar rates were observed in different studies (2.08%, 33.3%, and 64.58% and 15.4%, 56.4%, and 28.2%, respectively) [4,15,16].

### Conclusions and Recommendation

In this study, only 6.7% of doctors had good knowledge, so we recommend increasing the level of awareness and knowledge of SSI among medical interns, residents, and specialists in KAUH by providing more courses and sessions.

This study was subject to some potential limitations. It was carried out in a single center. Therefore, to get a global result, we recommend the initiation of future multicenter studies.

## Appendix

Multimedia Appendix 1 Questionnaire-based survey regarding awareness and level of knowledge about SSI and risks factors for wound infections among 119 physician respondents with answers to SSI-related questions in King Abdul-Aziz University Hospital between January and June 2018.

## Abbreviations

CDC: Centers for Disease Control and Prevention
HCAI: health care–associated infection
KAUH: King Abdulaziz University Hospital
SSI: surgical site infection
