# Supplementary material for: Awareness and Level of Knowledge About Surgical Site Infections and Risks of Wound Infection Among Medical Physicians in King Abdulaziz University Hospital: Cross-Sectional Study
Source: Interact J Med Res. 2019 Mar 6;8(1):e12769. doi: 10.2196/12769 (PMC6425309; doi:10.2196/12769)
Supplement: Multimedia Appendix 1 [file ijmr_v8i1e12769_app1.pdf]

Questionnaire-based survey regarding awareness and level of knowledge about SSI and risks factors for wound infections among 119 physician respondents with answers to SSI-related questions in King Abdul-Aziz University Hospital between January and June 2018.

Questions	Answers	Statistics, n (%)
Q1: The United States Centers for Disease Control and Prevention (CDC) has developed criteria that define SSI as an	(A) Infection related to an operative procedure that occurs at or near the surgical incision within *14 days* of the procedure; (B)Infection related to an operative procedure that occurs at or near the surgical incision within *60 days* of the procedure; (C) Infection related to an operative procedure that occurs at or near the surgical incision within *90 days* of the procedure; (D)Infection related to an operative procedure that occurs at or near the surgical incision within *30 days* of the procedure, or within *1 year* if prosthetic material is implanted at surgery.	66 (55.5)<br>19 (16.0)<br>6 (5.0)<br>28 (23.5)
Q2: SSIs are classified into incisional SSIs, which can be superficial, deep or organ/space SSIs. Superficial SSI means	(A)Infection involving the epidermis and dermis layers only; Infection involving both only the skin and subcutaneous tissue; (B) Infection involving fascial and muscle layers; (C) Infection involving internal organs manipulated during operation	49 (41.2)<br>60 (50.4)<br>6 (5.0)<br>4 (3.4)
Q3: Which is true about SSI classification:	(A) Deep incisional SSI is more common than superficial incisional SSI and organ/space SSI; (B) Deep organ SSI occurs within 60 days after operation; (C) Superficial incisional SSI accounts for more than half of all SSIs; (D) Superficial incisional SSI occurs within 14 days after operation	17 (14.3)<br>12 (10.1)<br>70 (58.8)<br>20 (16.8)
Q4: One of the most common isolated organisms in SSI is:	(A)*Staphylococcus aureus;* (B) *Streptococcus pyogenes*; *Escherichia coli*; (C) A+C	61 (51.3)<br>6 (5.0)<br>5 (4.2)<br>47 (39.5)
Q5: The best time for administrating prophylactic antibiotics is:	(A)Within 60 minutes prior to surgery; (B) Within 90 minutes prior to surgery; (C) Within 120 minutes prior to surgery; (D) Within 180 minutes prior to surgery	93 (78.2)<br>13 (10.9)<br>13 (10.9)

		0 (0.0)
Q6: Chances of developing SSI are:	(A) 1-3%; (B) 3%-5%; (C) 10%-5%; (D) 15%-20%	30 (25.2)<br><br>50 (42.0)<br><br>35 (29.4)<br><br>4 (3.4)
Q7: All the following pre-operative antibiotics are commonly used except:	(A)Cefazolin; (B) Cefoxitin; (C) Vancomycin; (D) Fidaxomicin	9 (7.6)<br><br>11 (9.2)<br><br>52 (43.7)<br><br>47 (39.5)
Q8: Which statement is correct about wound classification:	(A) Wound created in herniorrhaphy is considered as a clean-contaminated wound; (B) Appendiceal abscess is considered as a contaminated wound; (C) Clean-contaminated wound is defined as an incision under sterile condition; entrance of a hollow viscus with no active infection (D) Bowel obstruction with enterotomy and spillage of contents is considered as a dirty wound	6 (5.0)<br><br>32 (26.9)<br><br>58 (48.7)<br><br>23 (19.3)
Q9: Which one of these risk factors is LEAST associated with SSI:	(A)Prolonged pre-operative stay; (B) Hairy skin; (C)Poor postoperative glycemic control; (D) Type of wound	34 (28.6)<br><br>55 (46.2)<br><br>16 (13.4)<br><br>14 (11.8)
Q10: Complications of SSI include which of the following:	(A)Increased cost of care; (B) Death; (C) Fistula formation; (D) All of the above	6 (5.0)<br><br>6 (5.0)<br><br>7 (5.9)<br><br>100 (84.0)
Q11: The CDC[b] recommendations for the prevention of SSI include which of the following:	(A)Pre-operative showering with antimicrobial soaps; Blood glucose target level of less than 250 mg/dl; (C)Maintaining mild hypothermia; (D) Advising patients to shower at least 1 day prior to surgery	41 (34.5)<br><br>45 (37.8)<br><br>10 (8.4)<br><br>23 (19.3)

Q12: Infected wounds can exhibit which one of these presentations:	(A)Sweet smell; (B) Purulent pus; (C) Normothermia; (C) Painlessness	5 (4.2)<br>106 (89.1)<br>6 (5.0)<br>2 (1.7)
Q13: Prophylactic antibiotics are discontinued after surgery within:	(A) 4 to 8 hours; (B) 12 to 18 hours; (C) 24 to 48 hours; (D) 72 to 96 hours	22 (18.5)<br>28 (23.5)<br>56 (47.1)<br>13 (10.9)
Q14: Regarding hair removal for surgical patients, when is the best time:	(A)Just prior to surgical incision; (B)The night prior to surgery; (C) 2 hours prior to surgery; (D)30 minutes prior to surgery.	60 (50.4)<br>26 (21.8)<br>15 (12.6)<br>18 (15.1)
Q15: Regarding hair removal for surgical patients, it's best done by:	(A)Shaving; (B) Clipping; (C) Waxing; (D)Electrolysis	73 (61.3)<br>27 (22.7)<br>8 (6.7)<br>11 (9.2)
Q16: Which one of these factors impairs wound healing:	(A)Steroid use; (B) Hyperthermia; (C) Exposure to water; (D) Protein-rich food	99 (83.2)<br>5 (4.2)<br>10 (8.4)<br>5 (4.2)
Q17: In assessing nutritional status for a surgical patient, which statement is correct:	(A)Serum albumin level is the most commonly used marker to assess nutritional status; (B)Serum magnesium is a preferred marker over serum albumin; (C) Assessing the patient through inspection and further examination should be enough; (D)Poor nutritional status is not considered as a risk factor for SSI.	83 (69.7)<br>11 (9.2)<br>20 (16.8)<br>5 (4.2)
Q18: Based on World Health Orgainzation, the fourth step in hand hygiene technique is:	(A)Rub hands palm to palm; (B) Rotational rubbing, backwards and forwards with clasped fingers of right hand in left palm and vice versa; (C) Palm to palm with fingers interlaced; (D) Backs of fingers to opposing palms with fingers interlocked.	10 (8.4)<br>54 (45.4)<br>24 (20.2)<br>31 (26.1)
Q19: The first step in surgical scrubbing is:	(A)Ensure that your sleeves are at least two to three inches above your elbows; (B)Remove any watches and rings from your hands; (C) Open out your gown pack onto a clean table, only grabbing the outermost edges to maximize the sterile field; (D)Adjust water flow and temperature.	12 (10.1)<br>83 (69.7)<br>19 (16.0)<br>5 (4.2)
Q20: The purpose of pre-operative skin cleansing is:	(A)To achieve a good-looking skin; (B)To reduce risk of skin cancer; (C) To achieve a faster operation; (D)To reduce the burden of skin flora, thus reducing risk of SSI	11 (9.2)<br>6 (5.0)<br>6 (5.0)<br>96 (80.7)

a
